# Novel Parameter in Pre-Surgical Orthodontic Preparation: A Retrospective Study on the Role of the Upper Incisor Position and a Morphological Evaluation of the Anterior Nasal Spine

**DOI:** 10.3390/jcm13082346

**Published:** 2024-04-18

**Authors:** Ornella Rossi, Giovanna Perrotti, Massimo Del Fabbro, Tiziano Testori

**Affiliations:** 1 Department of Biomedical Surgical and Dental Sciences, University of Milan, 20122 Milan, Italy; massimo.delfabbro@unimi.it (M.D.F.); tiziano.testori@unimi.it (T.T.); 2 Private Practice, 22100 Como, Italy; giovanna.perrotti@lakecomoinstitute.it; 3 Department of Implantology and Oral Rehabilitation, Dental Clinic, IRCCS Ospedale, Galeazzi-Sant’Ambrogio, 20157 Milan, Italy; 4 Department of Periodontics and Oral Medicine, School of Dentistry, University of Michigan, Ann Arbor, MI 48109, USA; 5 Department of Oral Medicine, Infection and Immunity, Harvard School of Dental Medicine, Boston, MA 01451, USA

**Keywords:** ANB angle, anterior nasal spine, multiplanar cephalometry, facial aesthetic, facial attractiveness, orthognathic surgery

## Abstract

The position and inclination of the incisors play a crucial role in achieving optimal outcomes in orthodontic and orthognathic surgical treatment, given their impact on facial aesthetics. **Background/objectives**: Due to numerous distorting factors that affect the reliability of the ANB angle, the aim of the present work is to evaluate a more constant parameter over time, the anterior nasal spine (ANS), and explore whether aligning the incisal margin of the upper incisors with the anterior nasal spine could be a reliable indicator for achieving appropriate labial support in pre-surgical orthodontic preparation. **Methods**: From a pool of 500 cone beam computed tomography (CBCT) scans, 50 CBCT examinations displaying a Class 1 skeletal pattern (ANB = 2° ± 2°) with an intermediate (3.2–4 mm) or mixed (4–6 mm) sagittal maxillary position (MX), as determined by the 3D multiplanar total face approach (TFA), were selected and compared with CBCT examinations randomly chosen from the initial pool. Moreover, 12 landmarks were identified, and measurements were automatically obtained, using software, and recorded. Mean and standard deviation values were calculated for each sample. A comparison was made between the two samples, aligning the results with the morphological analysis of the anterior nasal spine and the sagittal position of the upper maxilla. **Results**: In Class 1 subjects, the distance between the incisal margin and the plane passed in relation to the anterior nasal spine should range between −1 mm and 1 mm, aligned with or slightly ahead of the anterior nasal spine or slightly ahead of this limit. **Conclusions:** The anterior nasal spine can serve as a reliable reference point for planning the position of the upper incisors, with excessive proclination or retroclination from this reference point deemed unacceptable.

## 1. Introduction

Orthodontic preparation plays a critical role in the success of orthognathic surgical treatment, ensuring the proper positioning of teeth within the periodontal framework relative to the underlying skeletal bases and contributing to the patient’s aesthetic satisfaction. During the pre-surgical phase, the orthodontist eliminates any dental compensation and adjusts the teeth, according to bone and dental references, to the proper position, creating a pre-surgical dental discrepancy equivalent to a skeletal discrepancy [1]. Planning the position and inclination of the incisors is crucial for achieving optimal orthodontic and orthognathic surgical treatment outcomes, as they significantly impact facial aesthetics [2]. The position of the central upper incisors directly influences the patient’s facial profile and labial support, posing a challenge during orthodontic treatment [3]. The relationship between the incisors, facial soft tissues, and bone bases guide the pre-surgical orthodontic phase of teeth decompensation, and cephalometric values assist the clinician in determining the need for dental extractions and the type of anchorage to adopt, in conjunction with dental casts. Correct cephalometric analysis and management of the position of incisors in orthodontic planning are becoming essential and dogmatic issues [4].

The cephalometric reference value for an upper incisor is the spino-incisal angle, which is formed between the long axis of the upper incisor and the bi-spinal plane of the PNS–ANS (posterior nasal spinal plane, anterior nasal spinal plane). In regard to the Class 1 skeletal pattern, a natural compensatory mechanism exists to address sagittal skeletal discrepancies that develop during growth [5,6]. Depending on the severity of the dysmorphosis, the inclinations of the upper incisor can vary significantly, leading to potential periodontal implications [7]. This underscores the importance of restoring normal incisor values, not only for correcting the skeletal class during surgery, but also for preserving periodontal health [8]. The concept of modulating a surgical intervention is contingent upon the incisal position and the established objectives. Bone and periodontal conditions permitting, achieving normal values may involve accentuating the overjet pre-surgically and surgically displacing the bone bases to a greater degree, resulting in a more significant aesthetic impact [9]. The compromises must be less accepted in patients with thin biotypes, reduced bone tissue thickness, and in the presence of periodontal problems [8]. Furthermore, in cases of severe vestibular inclination of the incisor group (which requires correction below 90°), even slight crowding (2–3 mm) and an accentuated Spee curve, necessitate extractions to establish the correct incisal position and inclination. Based on these considerations, the aim of the present study is to evaluate whether the position of the incisal margin of the upper incisors, in line with the nasal spine, is a reliable indicator for achieving appropriate labial support in pre-surgical orthodontic preparation.

## 2. Materials and Methods

From a pool of 500 CBCT (cone beam computed tomography) exams, two samples were selected for evaluating the position of the incisal margin relative to the anterior nasal spine and for assessing the relationship between the maxillary position and the length of the anterior nasal spine. Only cases meeting the inclusion and exclusion criteria were included in the study. The inclusion criteria comprised individuals with complete dentition or partial compromise, but with all the elements in the anterior region present (from canine to canine). Excluded from the study were exams where the patient’s head position was not in natural head position and subjects where the columella of the nose was not captured in the X-ray scan.

According to traditional 2D cephalometry, only 145 cases were classified in regard to the Class 1 skeletal pattern, based on the angular value of the Steiner ANB 2° ± 2°, and they were, in turn, analyzed with 3D multiplanar cephalometry and divided by sex. From this subset, 50 CBCT examinations were divided equally into 2 groups of 25 CBCTs, according to sex, for the first sample. The selection criteria for this sample included individuals with a Class I skeletal pattern (ANB = 2° ± 2°); an intermediate (3.2–4 mm) or mixed (4–6 mm) sagittal maxillary position, determined by multiplanar cephalometric analysis; a spino-incisal angular value within the normal range 110° ± 5°, as determined by 3D cephalometric analysis; a nasolabial angle value within the normal range: M (male) 98.7°–114.1° and F (female) 96.7°–110.3°. The second sample comprised 200 CBCT examinations, divided equally by sex and randomly selected from the initial pool of 500 CBCTs meeting the inclusion criteria and that had not been included in the first sample.

The consent of the ethics committee was not necessary to carry out the experimental protocol, since the DICOM (Digital Imaging and Communications in Medicine) data were acquired from radiographic examinations already prescribed for other reasons of dental relevance. The DICOM files were then uploaded to the Materialise Simplant Ortho O&O software (version 22.0, Materialise Co., Leuven, Belgium). All the axial, coronal, and sagittal images, and 3D renderings of the scanned structures were obtained for each sample. The field of view (FOV) was sufficiently large to encompass the entire maxillofacial region, from the glabella (G), the most prominent point of the frontal bone above the naso-frontal suture between the two eyebrow arches, to the menton (Me), the posterior point of the chin symphysis.

**Figure 1:**
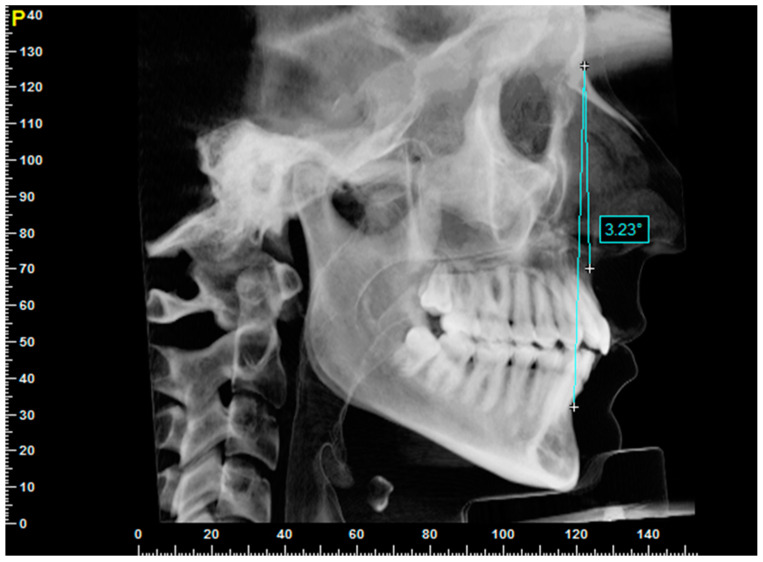
Ray-sum reconstruction of latero-lateral teleradiography from a CBCT examination.

The subjects included in the first sample underwent a double cephalometric assessment: the angular value of the ANB was calculated based on the latero-lateral projection, obtained by synthesizing the CBCT scans using the ray-sum technique (Figure 1), developed by the software. These subjects, classified with a Class 1 skeletal pattern based on the ANB values, were then analyzed with regard to the maxillary position, using the 3D multiplanar cephalometric analysis method, associated with the TFA module [10]. A second specific module has been created to analyze the superior incisal group.

**Figure 2:**
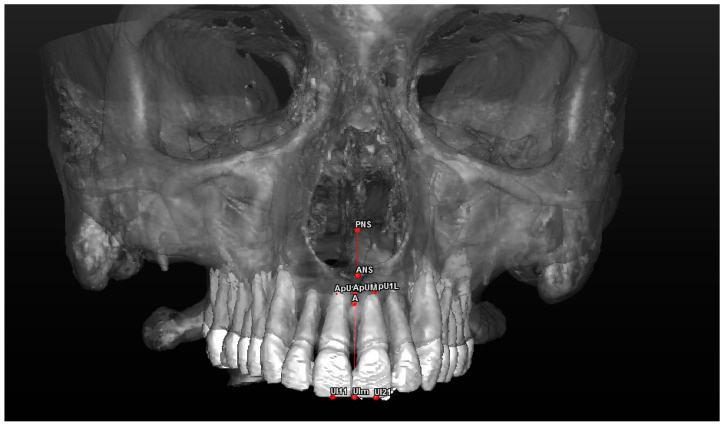
A 3D reconstruction of the upper jaw and dental arch, with a frontal view of the landmarks used in the study.

**Figure 3:**
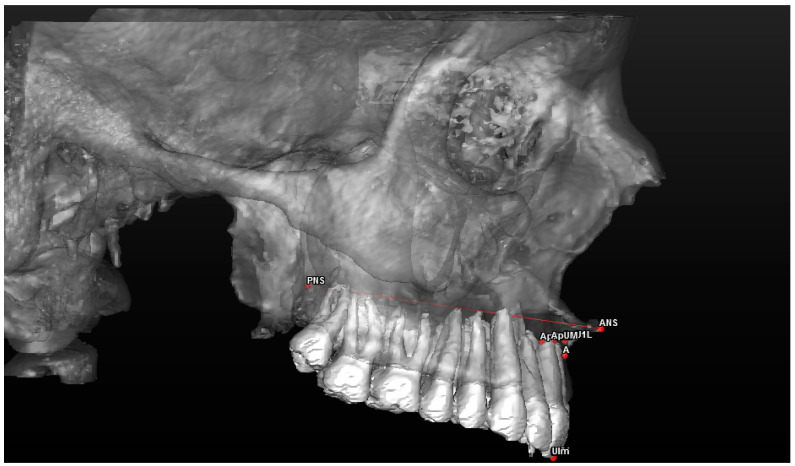
A 3D reconstruction of the upper jaw and dental arch, with a profile view of the landmarks used.

**Figure 4:**
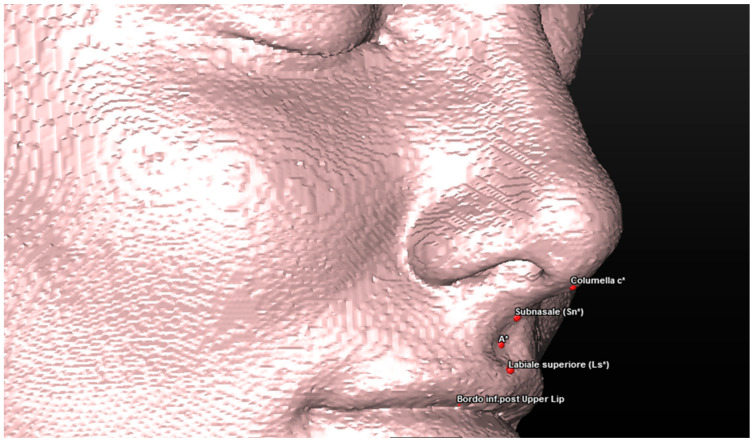
A 3D reconstruction of the patient’s soft tissues, with a view of the landmarks used.

Cephalometric analysis includes a series of cephalometric points (landmarks), 3 reference planes and 4 construction planes. The multiplanar reconstruction (MPR) system, as part of the software, was used to identify the landmarks in the 3D analysis. Image enhancement tools and maximum zoom capabilities were utilized to mark each point with certainty and accuracy across all three spatial planes. The landmarks used in our 3D cephalometric system consisted of 12 points, as follows:

- Seven landmarks for the dental and skeletal evaluation (shown in Table 1—Figure 2 and Figure 3);
- Five landmarks for the soft tissue aesthetic evaluation (shown in Table 2—Figure 4).

From the dental landmarks (U11, U21, ApU1R, and ApU1L), two median points were calculated directly by the software: UIm (the midpoint in the margins of the incisors, U11 and U21) and ApUM (the midpoint in the apexes of the incisors, ApU1R and ApU1L). These points allowed for the tracing of a straight line passing between the UIm and ApUM, the true median axis of the central incisors, defining the “Superior Incisal Axis”. Additionally, a straight line through the ANS and PNS bone landmarks was drawn, termed the “Spinal Axis”. The analysis involved angular and linear measurements derived from the distance between the cephalometric points and planes. The equation that is used to derive the distance separating any point *P* = (*x*_0_, *y*_0_, *z*_0_) from a plane is:

$$d(\pi ,P)=\frac{\left|ax_{0}+by_{0}+cz_{0}+d\right|}{\sqrt{a^{2}+b^{2}+c^{2}}}$$

**Figure 5:**
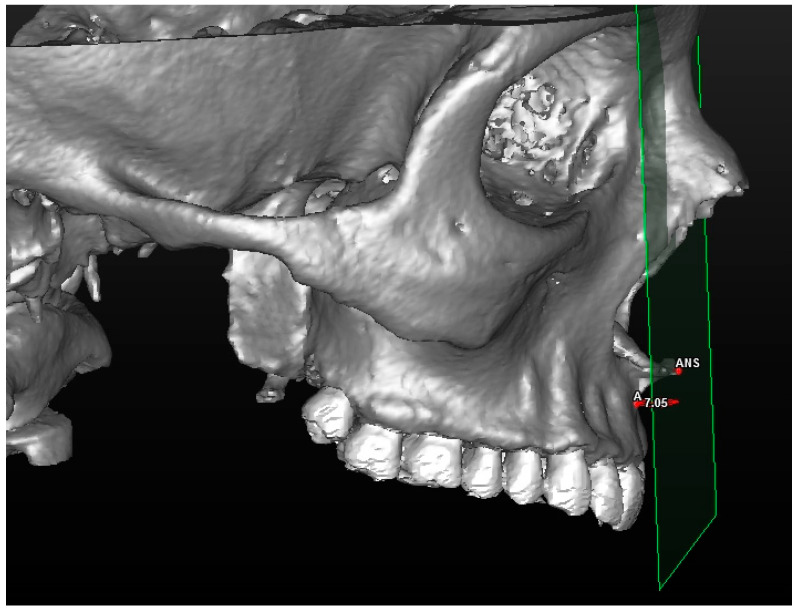
Evaluation of the sagittal skeletal position of the maxilla. The anterior facial plane (ATP) is the plane passing through the ANS and parallel to the coronal plane of reference.

**Figure 6:**
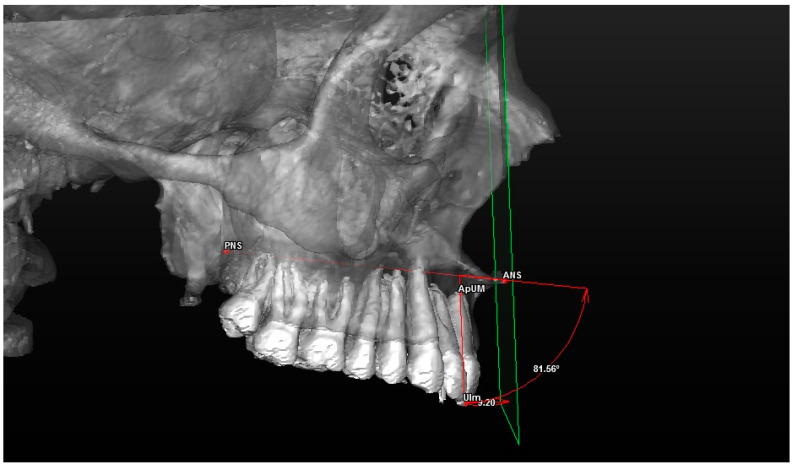
Evaluation of the dental component in profile (the spino-incisal angle: the angle between the spinal axis and the incisal axis).

**Figure 7:**
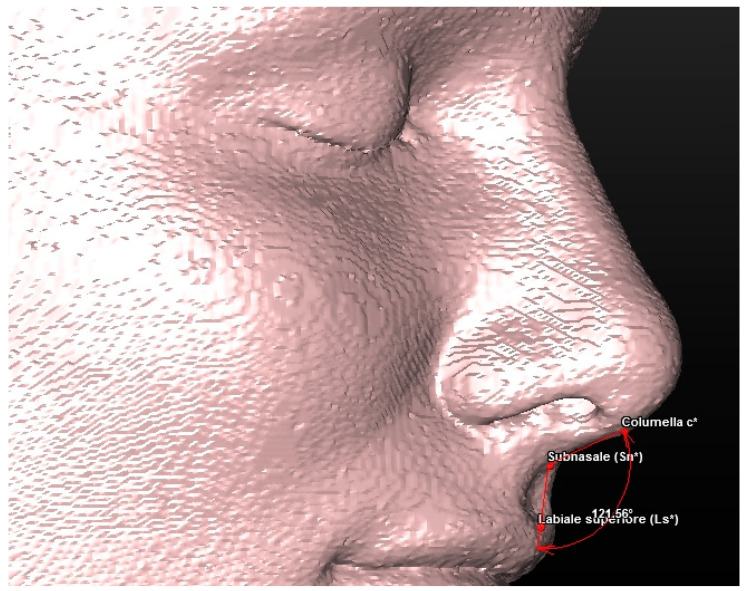
Soft tissue evaluation: nasolabial angle.

**Figure 8:**
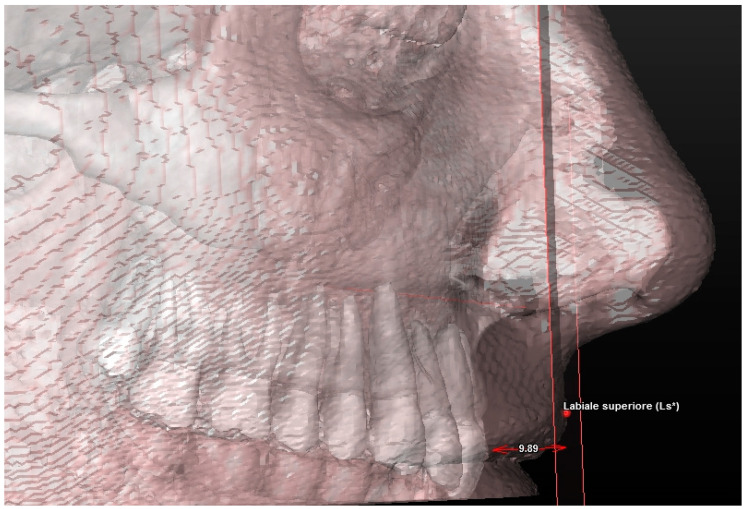
Soft tissue assessment: the upper lip height. The upper lip plane (ULp) is the plane passing through the Ls and parallel to the coronal plane of reference. * Nasolabial Angle is the angle formed between Columella (C), Subnasale (Sn) and Superior Labial (Ls).

**Figure 9:**
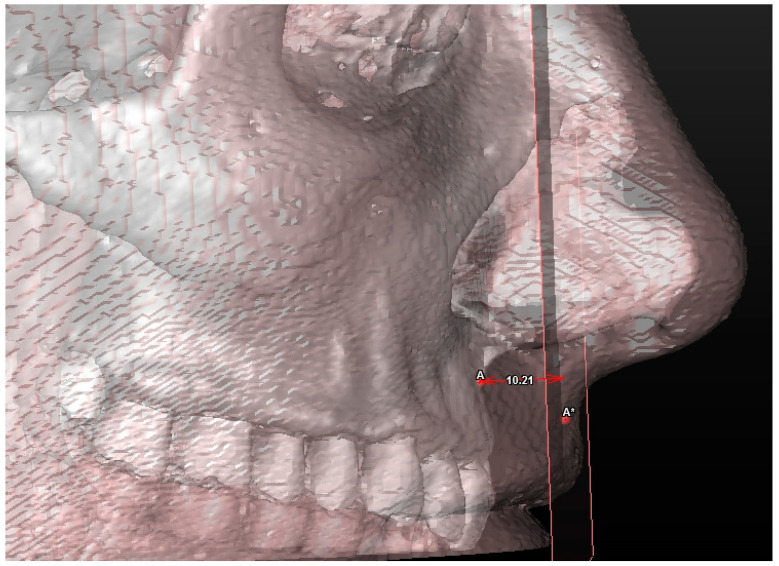
Soft tissue evaluation: coronal lip thickness. A* plane (A*p) is the plane passing through A* and parallel to the coronal plane of reference.

The 3D cephalometric analysis involving the first sample of 50 CBCT examinations included the calculation of the following seven measurements (Table 3):

(1) Maxillary sagittal position (MX) and anterior nasal spine length (A-ATP) (distance) (Figure 5);
(2) Spino-incisal angle (ANS–PNS to ApUM–Ulm) (angle) (Figure 6);
(3) Position of the upper incisal margin (Ulm–ATP) (distance);
(4) Nasolabial angle (Col–Sn–Ls) (angle) (Figure 7);
(5) Upper lip height (Sn–Ls) (distance) (Figure 8);
(6) Coronal lip thickness (BLs–Ulm) (distance) (Figure 9);
(7) Apical lip thickness (A–A’p) (distance).

The second sample, comprising 200 randomly selected cases, divided according to sex into 2 groups of 100, was utilized to assess the MX equating to the length of the anterior nasal spine (the distance between point A, the base of the anterior nasal spine, and the ANS, the apex of the anterior nasal spine). The cases were analyzed using a single analysis.

The measurement values were automatically obtained from the software and stored in CSV format, exported, and organized in a customized Excel folder. The statistical analysis involved calculating the mean and standard deviation of the two samples, analyzed individually.

## 3. Results

### 3.1. Evaluation of the Position of the Upper Incisal Margin

The first sample was analyzed according to the 3D cephalometric method. The mean and standard deviation were calculated for each measurement, and the results were divided according to sex (Table 4, Table 5 and Table 6): one male case and three female cases fell within the “Intermediate” value range for the maxillary sagittal position (A–ATP). The remaining cases, in both male and female groups, fell within the “Mixed MX” value range for the 3D multiplanar cephalometric analysis.

Only two cases (one male and one female) exhibited a slightly decreased spino-incisal angle (ANS–PNS to ApUM–Ulm), measuring 104.62° and 104.2°, respectively, compared to the normal value (110° ± 5°).

Only two cases (males) showed a distance greater than 1 mm between the incisal margin and the plane passing to A (Ulm–ATP), constituting 8% of the male group and 4% of the total sample. In females, no cases presented a discrepancy in the UIm–ATP measurement greater than 1 mm, but eight cases had values <0 (32%).

Only four cases, two females and two males, exhibited nasolabial angle (Col–Sn–Ls) values outside the normal range (males 98.7°–114.1°, females 96.7°–110.3°).

The values obtained for the height of the upper lip (Sn–Ls), the thickness of the coronal lip (Bls–Ulm), and the thickness of the apical lip (A–A’p) varied from the normal values typically reported in the literature, with only some cases falling within the normal range [11].

### 3.2. Morphological Evaluation of the Anterior Nasal Spine

The measurement of the MX was conducted on the second sample, which was compared to the length of the anterior nasal spine (A–ATP).

The mean and standard deviation were calculated for both male and female groups (4.95 ± 1.86 mm for males; F 4.67 ± 1.46 mm for females). The cumulative average was 4.8 ± 1.53, corresponding to the values considered “normal” in the first sample, assessed for the position of the upper incisal margin.

## 4. Discussion

Facial aesthetics is one of the main reasons patients seek orthodontic and surgical treatment, representing a key treatment objective, alongside achieving stable and functional occlusion [12]. While treatment planning is traditionally focused on the occlusal relationship, recent years have seen increased emphasis on achieving optimal facial profile harmony [13]. It has already been stated that correct occlusion does not always equate to a desirable facial profile and orthodontic treatments solely adhering to cephalometric standard values may not necessarily fulfill aesthetic principles [14]. In the assessment of facial aesthetics, it is crucial not only to evaluate occlusion and dental alignment, but also to consider the relationship between the skeleton and soft tissue [15]. The soft tissue profile, including the nasolabial and labio-mental zones, has received significant attention in recent years [16]. The maxillary incisors, with their labiolingual inclination and anteroposterior position, play a pivotal role in determining profilometric aesthetics [17,18].

Various cephalometric assessments have been performed to enhance the prediction of the correct position of the maxillary incisors, including Andrews’ analysis, which uses the patient’s forehead as a reference point but focuses solely on the anteroposterior position [12]. Cao examined the effect of labiolingual inclination and the anteroposterior position of the incisors, finding that while the anteroposterior position of the incisors is important for facial harmony, slight forward displacements of the incisors do not compromise the aesthetic profile “in smile”, whereas incorrect labiolingual inclinations can easily ruin the profilometric aesthetic harmony. However, this study’s limitation was its focus solely on Asian subjects [19].

An incorrect labiolingual inclination can result from several factors, including torque loss or incomplete expression. Gioka and Eliades [20] suggest prescription with high torque, to compensate for natural incomplete expression and tailored clinical needs; however, this is not indicated in cases of non-extractive crowding, where the upper incisors have an inclination very close to the desired angle [21].

In implant-prosthetic rehabilitation in the anterior region, both oral surgeons and prosthodontists face the challenge of determining the optimal position for the central incisors. In addition to the spasmodic search for structural perfection in regard to the prosthetic artifact, based on age, sex, and facial morphology, only the vertical position of the maxillary incisors was evaluated. The vertical position is determined by restoring the correct phonetic function, especially for labial-type sounds, and considering their relationship with the lips, based age and sex [22]. However, even within the realm of prosthodontics and rehabilitation, the correct position on the sagittal plane of the upper incisors to ensure proper labial support has not been investigated.

This study aimed to establish a new reference point for the ideal position of the upper incisors in relation to the correct nasolabial angle, which determines good upper labial support. The ANB angle is a simple and immediate evaluation method, and it has always been considered the reference point for orthodontic surgical treatment planning [23]. While a correct ANB angle (2° ± 2°) indicates a patient in the first skeletal class, it does not always correlate with patient satisfaction [16]. Furthermore, the ANB angle has been extensively criticized in the literature, due to discrepancies often detected between the measured value via cephalometry and the effective maxillary sagittal relationship [23]. Several factors have been identified that can influence the ANB angle, particularly the position of the nasion point, both sagittally and vertically, as demonstrated in Binder’s studies [24]. If the nasion point shifts forward, the ANB angle value decreases; although the actual relationship between the upper maxilla and the mandible remains unchanged. Conversely, a short anterior cranial base results in an increased ANB angular value; although the maxillary ratio remains unchanged [25]. Based on these considerations, the position of the incisors with respect to A and B points in orthodontic, orthodontic surgical, and prosthetic implants treatment planning, may vary among patients. From these considerations, it is necessary to establish a new fixed reference point, which involves minimal variation over time.

According to the studies performed by Enlow [26] on cranial structure growth, processes of apposition and resorption in the anterior maxillary area do not affect the apex of the anterior nasal spine, making it a constant referent point over time, which does not undergo remodeling or resorption processes even after the loss of anterior dental elements. However, there are no published articles in the scientific literature studying the anterior nasal spine, either anatomically or as a reference point for incisal position. According to the results obtained in this study, the distance between the incisal margin and the plane passed to the anterior nasal spine (Ulm–ATP) in subjects in the first skeletal class, except for two isolated male cases (4%), ranged between −1 mm and 1 mm in 96% of cases, and of the 96%, 24% (12 cases out of 50) was <0 and 72% (36 cases out of 50) was >0. The results suggest that the incisal margin of upper incisors should pass through or slightly ahead of the anterior nasal spine in regard to this limit, and an excessive proclination or retroclination from this limit is not acceptable. These results agree with a study by Schlosser [27], which demonstrated that retrusion of the upper incisors worsens the facial profile attractiveness of the subject as palatalization increases (their experimentation reached a maximum retrusion of 4 mm). The values obtained for the height of the upper lip differ to the normal values reported on average in the literature by Arnett [11], because Arnett’s analysis involved the use of the subnasal landmark and the stomion, while the present study used the “upper subnasal-labial” segment.

Finally, measurements of the lip thickness at both the coronal and apical levels, exhibit similarity among genders, but deviate from standard values [28] due to the distinction between 2D and 3D methodologies: in 2D assessments, parameters are recorded point-to-point, whereas in 3D assessments they are taken point-to-plane, ensuring a consistently perpendicular trajectory to the plane and, consequently, a more concise path. This purely mathematical explanation elucidates the variance from normative values [29].

To conclude, examining the anterior nasal spine as a novel reference for the incisor position emerges as a crucial variable for predicting how the upper incisors and, thus, the upper labial support is influenced by variations in the length of the anterior nasal spine.

## 5. Conclusions

The antero-posterior position of the upper incisors plays a fundamental role in orthodontic, maxillofacial surgery, and implant prosthetics treatment planning, since it modifies the aesthetic appearance of the patient’s soft tissues.

The nasolabial angle and the anterior nasal spine can be taken as reference points in planning the position of the upper incisors. According to the results of the present study, a slight protrusion of the upper incisors is preferable rather than their lingualization/retrusion regarding the perception of the anterior limit of the dentition.

These results could significantly influence both pre-surgical orthodontic preparation, accepting a greater forward inclination of the upper incisors in cases with long nasal spines, without resorting to extractions, distalization, or interproximal reduction, and also in implant-prosthetic rehabilitation.

Further studies and insights are needed to assess the relationship between the anterior nasal spine, the position of the upper incisors, and the support of the upper lip. For a suitable treatment outcome, both from an occlusal, functional, and aesthetic standpoint, it might be beneficial to provide a classification on the morphology of the anterior nasal spine, comparing a broader range of cases to obtain statistically significant results.

## Figures and Tables

**Table 1:** The 7 landmarks for the dental–skeletal evaluation.

	Point	Definition
Dental	U11	Midpoint of the incisal margin of the right central incisor
	ApU1R	Apex of the right central incisor
	U21	Midpoint of the incisal margin of the left central incisor
	ApU1L	Apex of the left central incisor
Skeletal	Point A	Backward point in the concavity of the upper maxilla between the ANS and the alveolar crest
	ANS (Anterior Nasal Spine)	Most advanced point in the profile of the upper maxilla at the level of the median palatine suture
	PNS (Posterior Nasal Spine)	Posterior and median point in the upper maxilla

**Table 2:** The 5 landmarks for the soft tissue aesthetic evaluation.

Point	Definition
Ls (Upper Labial)	Midpoint protruding more anteriorly than the upper labial vermilion
Sn (Subnasal)	Cutaneous midpoint of the encounter between the columella and the upper lip
Col (Columella)	Highest cutaneous midpoint of the columella at the meeting level between the nostrils
BLs (Back Edge of the Upper Lip)	Posterior–inferior point in the upper lip at the level of the incisal margin
A*	Cutaneous point of the corresponding subspinal point A

**Table 3:** Skeletal, dental, and soft tissue measurements.

	Measurement	Unit	Values Considered the “Norm”	Description
Skeletal	A–ATP (maxillary sagittal position = anterior nasal spine length)	Mm	3.2–6	Distance between the anterior facial plane and point A
Dental	ANS–PNS/ApUM–Ulm (spino-incisal angle)	Degrees	110 ± 5	Angle between the spinal axis and incisal axis
	Ulm–ATP (position of the upper incisal margin)	Mm	To search	Distance between the Ulm and the anterior facial plane
Soft tissue	Col–Sn–Ls (nasolabial angle)	Degrees	U 98.7–114.1 F 96.7–110.3	Angle between points columella–subnasal–upper labial
	Sn–Ls (lip height)	Mm	19–22	Distance between point Sn and Ls
	BLs–Ulm (coronal lip thickness)	Mm	U13.4–16.2 D10.8–14.4	Distance between BLs and Ulm
	A–A’p (apical lip thickness)	Mm	14–16	Distance between A and A*p

**Table 4:** Mean and standard deviation calculated for the first sample, divided according to sex.

Measurement	Values Obtained	Standard Values
Maxillary position	M 4.69 ± 0.55 mm F 4.73 ± 0.82 mm	Intermediate 3.2–4 mm Mixed 4–6 mm
Spino-incisal angle	M 109.9° ± 3.55° F 109.26° ± 3.37°	110° ± 5°
Position of the upper incisal margin	M 0.16 ± 0.65 mm F 0.1 ± 0.5 mm	-
Nasolabial angle	M 104.94° ± 3.64° F 105.32° ± 3.6°	M 98.7°–114.1° F 96.7°–110.3°
Upper lip height	M 14.98 ± 2.53 mm F 14.71 ± 2.67 mm	19–22 mm
Coronal lip thickness	M 10.99 ± 2.24 mm F 9.34 ± 2.07 mm	M 13.4–16.2 mm F 10.8–14.4 mm
Apical lip thickness	M 14.53 ± 1.75 mm F 13.21 ± 2.02 mm	14–16 mm

**Table 5:** Skeletal, dental, and soft tissue measurements for the male sample.

Patient (Male Sample)	1	2	3	4	5	6	7
	MX o A–ATP	ANSPNS su ApUM–Ulm	Ulm–ATP	Col–Sn–Ls	Sn–Ls	Bls–Ulm	A–A’p
1	5.06	113.62	0.16	102.15	14.14	12.23	16.34
2	4.59	107.59	0.75	104.33	20.44	9.92	15.46
3	5.67	104.62	1.6	98.47	10.07	10	13.28
4	4.16	111.1	0.32	110.81	15.7	10.15	12.23
5	4.33	105.81	0.43	109.94	11.97	9.93	13.75
6	4.23	107.65	−0.41	107.81	14.92	11.15	13.53
7	4.23	108.7	0.11	103.55	16.04	14.81	16.16
8	4.13	105.65	0.28	101.37	13.94	12.88	14.58
9	4.1	113.35	0.06	106.7	12.37	13.74	16.24
10	4.22	107.89	0.6	101.37	20.89	14.46	16.27
11	4.9	114.73	0.34	99.95	14.3	9.17	14.87
12	5.24	112.26	0.15	111.15	15.19	7.57	13.97
13	4.9	111.32	0.5	103.1	17.1	6	10.64
14	5.24	110.73	0.16	108.71	17.03	12.75	14.4
15	4.91	107.03	0.95	103.24	17.7	15.45	15.09
16	5.07	111.22	0.06	107.7	12.09	9.98	17.58
17	4.01	112.32	0.14	102.58	12.59	10.5	13.09
18	5.34	105.31	−1.75	105.17	12.48	11.93	17.65
19	4.15	113.41	0.23	108.3	16.52	12.1	15.13
20	3.96	114.9	−0.47	101.48	12.94	9.9	14.25
21	5.2	112.31	−0.89	101.24	12.29	9.91	14.9
22	4.77	104	0.4	107.6	9.01	11.66	14.65
23	5.79	100.07	0.36	103.4	11.57	13.5	12.11
24	4.85	109.87	0.84	115	9.09	12.72	13.3
25	4.43	103.47	−0.58	104.73	10.49	15.85	16.85

**Table 6:** Skeletal, dental, and soft tissue measurements for the female sample.

Patient (Female Sample)	1	2	3	4	5	6	7
	MX o A–ATP	ANSPNS su ApUM–Ulm	Ulm–ATP	Col–Sn–Ls	Sn–Ls	Bls–Ulm	A–A’p
1	4.85	110.67	0.64	109.3	15.69	9.75	14.18
2	5.15	105	0.01	104.03	14.96	13.31	13.44
3	4.11	106.13	−0.11	108.03	17.46	7.9	13.19
4	5.83	109.6	0.18	104.72	15.73	7.59	11.92
5	4.66	111.95	−0.8	104.59	11.17	9.18	8.48
6	4.01	110.05	0.32	103.69	14.07	11.19	14.68
7	4.02	105.81	0.78	100.7	14.98	11.31	14.13
8	4.44	106.65	0.19	109	16.65	8.57	13.35
9	4.05	114.96	−0.55	100.51	16.09	11	14.14
10	6	112.06	0.71	106.47	21.55	12.25	16.61
11	4.22	105.97	−0.66	107.6	15.6	6.51	13
12	4.66	113.26	0.4	109.53	14.12	10.84	13.39
13	6	110.7	0.07	102.67	14.42	8.82	14
14	4.99	109.78	0.33	110	14.01	9.01	12.8
15	3.29	108.82	0.03	103.44	9.54	9.54	14.64
16	3.82	108.28	0.22	103.52	15.99	7.62	10.95
17	5.98	107.77	−0.22	102.63	10.89	7.76	11.51
18	3.71	105.7	0.4	110.2	12.03	7.66	10.98
19	4.9	107.62	−0.79	107.88	11.47	5.18	12.03
20	4.13	113.93	−0.6	97.59	16.69	13.63	17.54
21	4.37	105.78	0.68	102.53	13.97	10.1	12.28
22	4.07	109.92	0.89	106	10.79	11.5	15
23	5.32	104.02	−0.38	115	7.15	12.18	12.76
24	5.9	110.02	0.19	105	12.68	15.92	19.57
25	5.76	100.93	0.52	114.95	10.19	14.47	13.34

## Data Availability

The data are available upon request to the corresponding author.
